# Protein-Protein Interactions in Alpha-Synuclein Biogenesis: New Potential Targets in Parkinson’s Disease

**DOI:** 10.3389/fnagi.2020.00072

**Published:** 2020-03-17

**Authors:** Sarah M. Hernandez, Elena B. Tikhonova, Andrey L. Karamyshev

**Affiliations:** Department of Cell Biology and Biochemistry, Texas Tech University Health Sciences Center, Lubbock, TX, United States

**Keywords:** Parkinson’s disease, alpha-synuclein, disease-causing mutations, protein-protein interactions, protein quality control, protein misfolding, translational control, neurodegenerative diseases

## Abstract

Parkinson’s disease (PD) is a debilitating neurodegenerative disorder defined by a loss of dopamine-producing neurons in the substantia nigra in the brain. It is associated with cytosolic inclusions known as Lewy bodies. The major component of Lewy bodies is aggregated alpha-synuclein. The molecular mechanism of alpha-synuclein aggregation is not known. Our conceptual model is that alpha-synuclein aggregates due to a dysregulation of its interactions with other protein partners that are required for its biogenesis. In this mini review article, we identified alpha-synuclein interactions using both current literature and predictive pathway analysis. Alterations of these interactions may be crucial elements for the molecular mechanism of the protein aggregation and related pathology in the disease. Identification of alpha-synuclein interactions provides valuable tools to understand PD pathology and find new pharmacological targets for disease treatment.

## Introduction

Aggregated aSyn is found within Lewy bodies present in the cytoplasm of neurons from patients with synucleinopathies. Parkinson’s disease (PD), Lewy Body Dementia (LBD), and Multiple System Atrophy (MSA) are all examples of synucleinopathies. PD is the second most common neurodegenerative disorder, after Alzheimer’s disease. It is classified by a loss in motor control due to the death of dopaminergic neurons as a result of aSyn aggregation and associated toxicity. PD is an age-related disease as it is mostly diagnosed in the elderly and its incidence increases exponentially in people over 60 years of age (Hindle, 2010; Rodriguez et al., 2015). Current therapies for PD are based on the supplementation of dopamine, however, this therapy is limited in duration and effectiveness (Verschuur et al., 2019). Alternative approaches are needed to develop new PD treatments and find markers for early disease detection. Discovering molecular mechanisms of aSyn biogenesis and its aggregation will certainly help to understand the mechanisms of PD and develop new therapies. Knowledge about aSyn biogenesis, including transcription, translation, translocation, modification, and degradation, will shine light into understanding why aSyn aggregates and causes disease.

## Alpha-Synuclein Biogenesis, Aggregation, and Spread of Pathology

**Figure 1:**
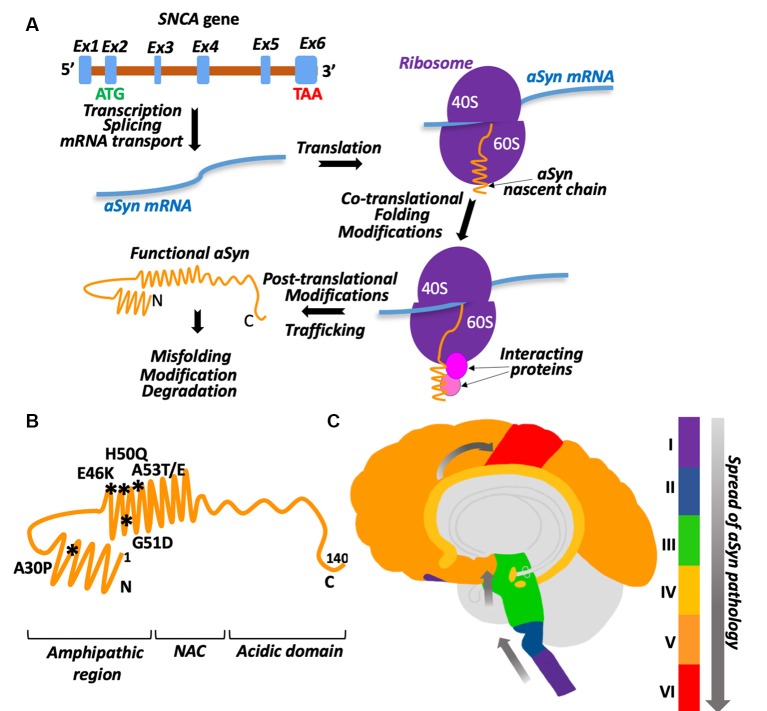
aSyn biogenesis and pathology. **(A)** Graphic illustration of aSyn biogenesis. SNCA gene is encoded on chromosome 4. Its transcript is composed of six predicted exons with a start codon (ATG) localized in exon 2 and stop codon (TAA) in exon 6. After mRNA splicing and transport into the cytosol, synuclein is translated into 15 kDa protein. Proper folding and necessary modifications occur co- and post-translationally with the help of chaperones and other proteins. Ex1–Ex6 are exons, shown as blue boxes on a pre-mRNA (brown), ribosomes are shown as two purple half-spheres (40S and 60S subunits), aSyn mRNA is a blue waved line, interacting proteins are depicted as spheres. See the text for details. **(B)** Schematic presentation of aSyn protein structural domains with the location of clinical mutations. Amino acid residues 1–60: N-terminal amphipathic region (clinical mutations are shown and their positions are marked by asterisks, the region contains ubiquitination and SUMOylation sites). Amino acid residues 61–95: non-amyloid beta component (NAC). Amino acid residues 96–140: C-terminal acidic domain (contains phosphorylation sites). **(C)** Schematic presentation demonstrating the spread of aSyn pathology in the brain with Parkinson’s disease (PD). Stage I: olfactory bulb/medulla (purple), stage II: pons (blue), stage III: midbrain (including substantia nigra-green), stage IV: limbic lobe (yellow), stage V: neocortex (orange), stage VI: primary sensory/motor cortices (red).

Alpha-Synuclein (aSyn) is a relatively small protein (~15 kDa) encoded by the SNCA gene (Figure 1A). The specific pathological feature of this protein is its ability to aggregate. The structure of aSyn consists of two alpha-helices followed by an unstructured, acidic C-terminal tail (Figure 1B). The first alpha-helix is positively charged and can bind to lipids due to the repeating KTKEGV motif allowing for the formation of an alpha-helical structure (Davidson et al., 1998; Zarbiv et al., 2014). The second alpha-helix contains the non-amyloid beta component (NAC) which is a hydrophobic region that is required for aggregation (Giasson et al., 2001; Du et al., 2003). There are two major types of pathogenic aSyn mutations seen in synucleinopathies. The first increases the dose of aSyn, which includes dinucleotide repeat variations in the promoter region and locus multiplications (Karimi-Moghadam et al., 2018). The second is missense point mutations. The missense point mutations identified so far include A30P, E46K, H50Q, G51D, A53E, and A53T (Krüger et al., 1998; Spira et al., 2001; Zarranz et al., 2004; Lesage et al., 2013; Khalaf et al., 2014; Pasanen et al., 2014). A30P is on the first alpha-helix, while the rest are on the second alpha-helix (Figure 1B). Missense point mutations have been shown to alter the aggregation propensity and kinetics of aSyn (Li et al., 2001; Greenbaum et al., 2005; Fares et al., 2014; Ghosh et al., 2014; Khalaf et al., 2014).

aSyn is found ubiquitously throughout the brain and the body, with the highest levels in the blood, central nervous system, and smooth muscles. In PD, aSyn pathology is not only seen in the brain but the gut as well and may be associated with a change in the gastrointestinal microbiota (Scheperjans et al., 2015). One hypothesis suggests that aSyn aggregates may travel from the gut to the brain through the vagal nerve and the olfactory bulb (Braak et al., 2003; Kim et al., 2019). In the early stages of PD, Lewy bodies are seen in the olfactory bulb and the medulla (stage I, Figure 1C). Then, these Lewy bodies spread to the brainstem and pons (stage II) and midbrain, which includes the substantia nigra (stage III). Next, pathology propagates to the limbic lobe (stage IV), neocortex (stage V), and eventually to the primary and motor cortices at late stages of the disease (stage VI; Beach et al., 2009). aSyn is transferred from one cell to another in a prion-like manner (Freundt et al., 2012; Helwig et al., 2016) most likely by exosomes (Emmanouilidou et al., 2010; Danzer et al., 2012). Interestingly, misfolded aSyn was found more likely to be secreted (Jang et al., 2010).

## Protein-Protein Interactions in aSyn Biogenesis

**Figure 2:**
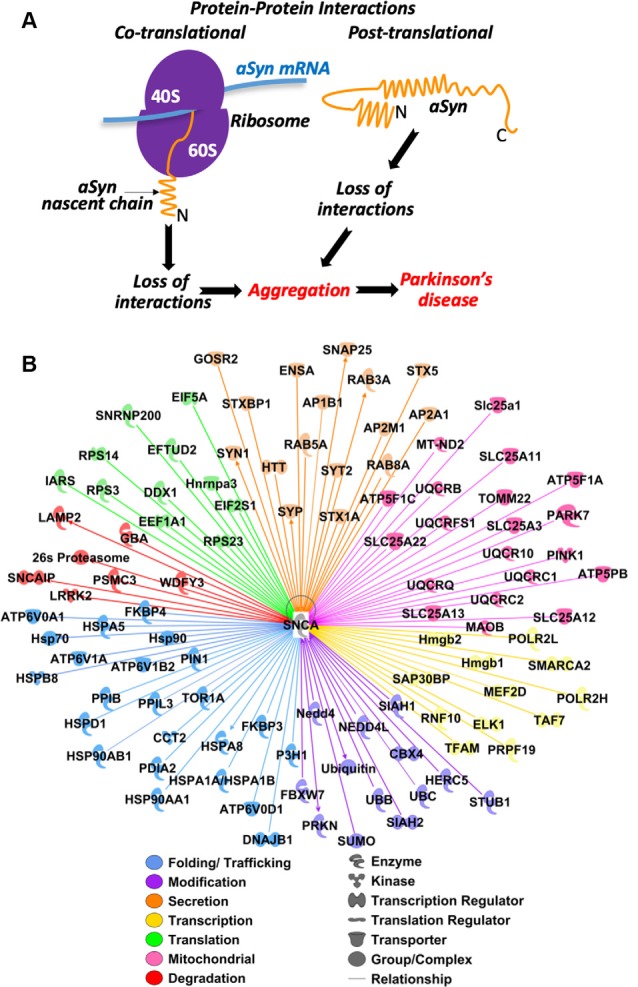
Predicted protein-protein interactions in aSyn pathology: hypothesis and bioinformatic analysis. **(A)** Conceptual model of aSyn pathology induced by loss of co-translational and post-translational interactions due to mutations in aSyn or omission of partners. **(B)** Prediction of aSyn protein-protein interaction during biogenesis. Ingenuity Pathway Analysis (IPA) software (Qiagen) was used to analyze possible aSyn interactions and pathways. aSyn gene SNCA was selected as the primary molecule (shown in the center). The initial analysis revealed 474 interacting proteins. The pathway was trimmed to only show proteins that are complexes, enzymes, kinases, phosphatases, groups, transcription regulators, translation regulators, transmembrane receptors, or transporters. Two-hundred and seventy-eight interacting proteins remained and were filtered based on their major functions. Functions examined are those involved in transcription (yellow), translation (green) folding/trafficking (blue), modification (purple), secretion (orange), mitochondrial (pink), and degradation (red). One-hundred and two interacting proteins remained. All filtering was done to specifically analyze the protein-protein interactions involved in aSyn biogenesis.

During their biogenesis, proteins form multiple interactions with other proteins for proper folding, modification, transport, regulation of activity, recycling, and degradation. Many of these interactions occur co-translationally when proteins are being synthesized on the ribosome (Karamyshev and Karamysheva, 2018). As demonstrated on several other proteins, alteration of these interactions can interfere with protein biogenesis and lead to multiple human diseases, including neurodegenerative disorder Frontotemporal Lobar Degeneration (FTLD; Patrick et al., 2011; Karamyshev et al., 2014; Nilsson et al., 2015; Vetter et al., 2016; Pinarbasi et al., 2018; Karamysheva et al., 2019; Tikhonova et al., 2019). We propose that dysregulation of aSyn interactions, particularly during translation, results in protein misfolding, aggregation, and finally in disease progression. Aggregation could be initiated by the loss of assistance during aSyn biogenesis induced by a mutation in aSyn or a loss of an interacting partner from the system (Figure 2A). Protein biogenesis is an important process in determining the vitality of a cell. Defective proteins can disrupt normal functioning, form aggregates, and even lead to cell death. aSyn protein-protein interactions can occur on a co-translational and post-translational level. Beginning at the early stage of translation, the nascent chain is already exposed to a variety of interacting partners. These partners include chaperones/chaperonins, translocating and targeting factors, modifying enzymes, and a number of others (Karamyshev and Karamysheva, 2018). These factors are essential for the proper folding, localization, and function of the new protein during its synthesis. Interacting partners can also affect protein biogenesis post-translationally. Here we show an aSyn protein-protein interaction network using Ingenuity Pathway Analysis (IPA, Qiagen). IPA predicts interacting pathways using data from current literature. The IPA in Figure 2B shows a complex network that features 102 interacting partners of aSyn. These interacting partners are highlighted in seven different clusters depending on their function, including transcription, translation, folding/trafficking, modification, secretion, mitochondrial-associated, and degradation (Figure 2B).

The transcription cluster is defined by having a role in regulating transcription, RNA synthesis, or RNA processing. There are 12 proteins in this category. Subunits H and L of RNA Polymerase II (POLR2H/L) are involved in RNA synthesis. Transcription factors ELK1 and A, RNF10, Hmgb1/2, MEF2D, TAF7, SAP30BP, and SMARCA2 are involved in transcription regulation. The ubiquitin-protein ligase PRPF19 is involved in RNA processing. Interactions with proteins involved in transcription might affect the expression level of aSyn leading to changes in the aggregation profile of aSyn protein.

The translation cluster is grouped by having a role in initiation, elongation, and assembly of the ribosome. There are 11 proteins in this category. DEAD-box protein DDX1, initiation factors (EIF) 2 subunit alpha and 5A are involved in initiation. tRNA synthetase IARS is involved in tRNA aminoacylation, EFTUD2 and EEF1A1 are elongation factors. Ribosomal proteins (RPS) S3, S14, and S23, ribonucleoproteins SNRNP200 and Hnrnpa3 are involved in ribosome assembly. Translation is the process by which new proteins are synthesized. Proteins involved in this process may be vital to understanding normal and pathogenic aSyn biogenesis.

The folding/trafficking group is categorized as any protein that assists in the folding, transport, and localization of proteins. There are 23 proteins in this category. HSP70 family members (HSPA5, HSPA8, HSPA1A/HSPA1B), HSP90 class A members 1 and 2 (HSP90AA1, HSP90AB1), HSP60 member 1 (HSPD1), and TriC/CCT subunit 2 (CCT2) are chaperones. HSPB member 8 (HSPB8) and HSP40 member B1 (DNAJB1) are co-chaperones. Proteins ATP6V0A1/D1 and ATP6V1A/B2 are subunits of V-type ATPase involved in the translocation of protons across the lysosomal, endosomal, and Golgi membranes. Isomerases FKBP3/4, PPIB, PPIL3, PDIA2, PIN1, hydroxylase P3H1, and ATPase TOR1A, are involved in protein folding and trafficking. Interactions with proteins presented in this group could have a great impact on aSyn biogenesis and pathological properties.

The modification cluster is defined as any protein involved in the addition of a group to proteins and includes 13 members of this pathway. E3 ubiquitin ligases HERC5, NEDD4, NEDD4l, PRKN, FBXW7, STUB1, and SIAH1/2, and ubiquitin B/C are involved in ubiquitination. SUMO and its ligase CBX4 are involved in SUMOylation. Modification of proteins is vital to their future function or survival.

The secretion cluster is categorized as proteins involved in the transport of proteins out of the cell and includes 16 proteins in this pathway. RAB3A/5A/8A, HTT is involved in microtubule-mediated transport and vesicle function, syntaxins STX5/1A, STXBP1, SNAP25, GOSR2, ENSA are involved in SNARE docking. SYP, SYT2, SYN1, are involved in vesicle trafficking. Adaptor proteins AP2B1, AP2A1, and AP2M1 are involved in clathrin-coated vesicles. The mechanism of aSyn secretion and propagation is still not clear. Finding the interacting partners in this pathway might help to shed light on how the disease progresses.

The mitochondrial cluster is defined as proteins involved in the homeostasis of the mitochondria. It includes 20 proteins with different functions and localizations in both the inner and outer membrane of the mitochondria. ATP synthase subunits ATP5F1A, ATP5F1C, and ATP5PB are present on the inner membrane. Amino acid transporters SLC25A1, SLC25A11, SLC25A12, SLC25A13, and SLC25A22 are located on the inner membrane and SLC25A3 is located on the outer membrane. Electron transport chain members MT-ND2, UQCRB, UQCR10, UQCRFS1, UQCRC1, UQCRC2, and UQCRQ are located on the inner membrane. MAOB is on the outer membrane and oxidizes mono-amines. PARK7 (DJ-1) is a redox-sensitive chaperone. PINK1 protects the cell from stress-induced mitochondrial dysfunction. TOMM22 is on the outer membrane and imports cytosolic pre-proteins into mitochondria. It is unclear how aSyn is transported into the mitochondria, but determining its interactions both inside and outside could help uncover how it creates dysfunctional mitochondria homeostasis. A potential relationship between mitochondrial dysfunction and aSyn aggregation is described in the recent review (Faustini et al., 2017).

The degradation cluster is grouped by any protein involved in the proteasomal or autophagy (lysosomal) degradation pathway and includes seven proteins. The 26S proteasome, PSMC3, and SNCAIP are involved in the proteasomal pathway. LAMP2, WDFY3, GBA, and LRRK2 are involved in the autophagy pathway. Undoubtedly, fine-tuning aSyn expression levels by interacting with ubiquitination factors and degradation machinery could have a significant influence on aSyn aggregation and subsequent degradation.

In addition to the IPA analysis, we completed a review of publications focusing on the proteins that are involved in aSyn folding, trafficking, modification, and aggregation.

## Folding/Trafficking and aSyn Aggregation

Chaperones are proteins that help with the folding of other proteins. They can interact co-translationally to assist with the folding of nascent polypeptide chains as they exit the ribosome. Chaperones can also act post-translationally to help prevent and reverse aggregation of proteins through either refolding or assisting in the degradation of the misfolded protein (Hartl et al., 2011). Several chaperones have been shown to associate with aSyn. Small heat shock proteins (sHSPs) αB-c and HSP27 (HSPB1) inhibit aSyn aggregation in a concentration-dependent manner. However, the increased rate of A53T aggregation makes sHSPs less effective at inhibiting aggregation due to an overwhelming amount of aggregates (Cox et al., 2016). HSP70 (HSPA8) together with co-chaperone HSP40 (DNAJB1), HSP110 family member Apg2 (HSPA4), and sHSPB5 (CRYAB) act as a disaggregase system to solubilize aSyn aggregates by promoting both fragmentation and depolymerization of fibrils (Duennwald et al., 2012; Gao et al., 2015). HSP90 normally functions as a negative regulator of heat shock factors, including HSP70. Treatment with Geldanamycin, an inhibitor of HSP90, prevents aSyn-mediated dopaminergic cell loss in *Drosophila*, despite the continued presence of Lewy bodies (Auluck et al., 2002). How is HSP90 affecting the disaggregase system in diseased patients? Brains of Lewy Body Dementia patients have higher levels of insoluble aSyn protein that are associated with higher levels of insoluble HSP90 and HDJ1 (DNAJB1) and soluble HSP70 (Cantuti-Castelvetri et al., 2005; Paleologou et al., 2009). These increased levels may decrease the disaggregase system, letting aSyn continue to aggregate. Direct interactions between mitochondrial chaperone DJ-1 (PARK7) and both monomeric and oligomeric forms of aSyn were reported. DJ-1 reduces the oligomerization of aSyn *in vitro*. In agreement, familial mutations in DJ-1 disrupt this interaction and fail to reduce aSyn oligomerization (Zondler et al., 2014). The chaperones proSAAS and DNAJB6 protect against aSyn-induced toxicity by preventing aSyn aggregation (Jarvela et al., 2016; Aprile et al., 2017). The chaperonin, TRiC/CCT, regulates the folding of 10% of cytosolic proteins and has been shown to counter toxic inclusions from mutant huntingtin, another protein prone to aggregation (Sontag et al., 2013). Mutant huntingtin exhibits a similar aggregation and pathology as aSyn, therefore, TRiC/CCT may also be involved in aSyn regulation. Chaperones HSPA8, DNAJB1, DJ-1, and TRiC/CCT subunit CCT2 were also identified *via* our IPA pathway. Overall, many chaperones are seen to associate with aSyn and reduce its aggregation. However, these interactions are distorted in diseased brains, leading to the accumulation of aSyn aggregation and subsequent toxicity.

## Role of Modifying Enzymes in aSyn Aggregation

Co-translational and post-translational modifications are crucial points of regulation of protein function. During biogenesis, aSyn is phosphorylated, acetylated, SUMOylated and ubiquitinated. These modifications can occur co-translationally when nascent chains are exiting the ribosomes, as well as post-translationally on mature proteins.

Phosphorylation is the addition of a phosphate group co- or post-translationally. Several studies demonstrated that phosphorylation of aSyn at residue S129 is increased with A30P and A53T mutants within Lewy bodies (Fujiwara et al., 2002; Kahle et al., 2002; Takahashi et al., 2003; Anderson et al., 2006; Waxman and Giasson, 2008). S129 is phosphorylated by kinases CK-1, CK-2, and G protein-coupled receptors (Okochi et al., 2000; Pronin et al., 2000; Ishii et al., 2007). Phosphorylation at S87 by kinase DYRK1A increases aSyn aggregation and potentiates cytotoxicity (Kim et al., 2006). Residues Y133 and Y136 are phosphorylated by tyrosine kinase, SYK, while Y125 is phosphorylated by both, SYK, and SRC family kinase, FYN (Ellis et al., 2001; Nakamura et al., 2001; Negro et al., 2002). aSyn does not oligomerize if it is first phosphorylated by SYK (Negro et al., 2002), emphasizing the clear connection between phosphorylation and the oligomeric state of aSyn protein. Y125 phosphorylation protects against neurotoxicity by preventing oligomerization. Phosphorylation at Y125 diminishes with age and is lower in patients with Lewy Body Dementia (Chen et al., 2009). Phosphorylation at Y39, S87, S129 decreases the affinity of the helix-2 for membrane surfaces so the binding potential is comparable to that seen with A30P and G51D mutants (Paleologou et al., 2010; Dikiy et al., 2016). Phosphorylation at Y39 by kinase c-Abl is correlated with the progression of PD and inhibition leads to increased degradation of aSyn (Dikiy et al., 2016). However, other studies have shown that S87, S129, and Y125 phosphorylation actually prevent fibrillization (Waxman and Giasson, 2008; Chen et al., 2009; Paleologou et al., 2010). All of these results support the hypothesis that phosphorylation significantly affects aSyn oligomerization and degradation.

Acetylation is the addition of an acetyl group to a protein either co-translationally or post-translationally. aSyn is acetylated on the N-terminus at sites M1, K6, and K10, though the enzyme responsible is still not known (Kang et al., 2012; de Oliveira et al., 2017). Acetylated aSyn has a more stable helix and increased lipid affinity, with a decrease in B-sheet propensity in regions with known familial mutations (Kang et al., 2012; Maltsev et al., 2012; Dikiy and Eliezer, 2014). Deacetylation of aSyn *via* deacylase SIRT2 increases aggregation and cytotoxicity *in vivo* through reduced clearance by the lysosome(de Oliveira et al., 2017).

SUMOylation and ubiquitination are both important modifications that occur post-translationally and are involved in regulating protein degradation. SUMOylation is the addition of small ubiquitin-like modifiers (SUMOs) to a protein to affect a protein’s structure and localization (Wilkinson and Henley, 2010). aSyn is SUMOylated at lysines in positions 6, 10, 12, 21, 23, 34, 45, 60 at the N-terminus, 80 in the NAC region, and 96 and 102 at the C-terminus by E3 SUMO ligases PIAS2, hPc2, and SUMO-1. They constitute 11 out of 15 total lysines found in aSyn (Krumova et al., 2011; Oh et al., 2011; Rott et al., 2017). SUMOylation by PIAS2 decreases aSyn ubiquitination and subsequent proteasomal degradation and SUMOylation by SUMO-1 is found associated in Lewy bodies (Kim et al., 2011; Rott et al., 2017). However, SUMOylation has also been found to prevent aSyn aggregation and protect the cell from cytotoxicity (Krumova et al., 2011; Shahpasandzadeh et al., 2014). SUMO is also seen in the IPA pathway.

Ubiquitination is site-specific and differs between soluble and filamentous forms of aSyn. Soluble aSyn is mainly ubiquitinated at lysines in positions 21 and 23, as well as 32 and 34. Filamentous aSyn is ubiquitinated at lysines in positions 6, 10, and 12 by NEDD4 ligase (Nonaka et al., 2005; Mund et al., 2018). Ubiquitination of aSyn mediated by the E3 Ubiquitin ligase CHIP reduces aSyn oligomerization, while chaperone BAG5 mitigates ubiquitination by CHIP (Kalia et al., 2011). Ubiquitination by SIAH1 promotes aggregation of aSyn (Lee et al., 2008). CHIP (STUB1) was also identified in the IPA pathway, along with ubiquitin.

## Degradation Pathways of aSyn

Protein degradation is an important factor to maintain a proper balance of protein proportions in the cell and a means to get rid of aberrant proteins and prevent protein aggregation. There are two major protein degradation pathways—proteasomal and lysosomal. As shown in Figure 2B, aSyn is capable of interacting with components of both degradation pathways. In lysosomal degradation, aSyn is lead to the lysosome by heat shock protein HSP70, which then delivers aSyn to LAMP-2A for internalization. Mutant aSyn and LRRK2 (protein associated with PD) obstruct the autophagy pathway through high-affinity binding to LAMP-2A (Cuervo and Wong, 2014). Knockout of lysosomal trafficking receptor LIMP-2 results in overexpression of aSyn, neuronal death, and serious neurological problems in mice, while overexpression of LIMP-2 increases clearance of aSyn through the lysosome (Rothaug et al., 2014). Mutations in lysosomal hydrolase, GBA1, are a strong risk factor for the development of PD. Loss of function GBA1 mutations increase levels of aSyn monomers and decrease the levels of aSyn tetramers by compromising lysosomal activity (Du et al., 2015; Kim et al., 2018). aSyn accumulation induces lysosomal dysfunction in both cell culture and in PD patients through the reduction of hydrolase activity. This effect does not occur when the hydrophobic portion of aSyn (NAC region) is deleted (Mazzulli et al., 2016). The addition of 3-MA, autophagy pathway inhibitor, greatly increases the steady-state levels of aSyn, suggesting that autophagy is the predominant degradation pathway for aSyn (Vogiatzi et al., 2008). LAMP-2A (LAMP2), dardarin (LRRK2), and GCase (GBA1), proteins that function in the lysosomal pathway, were identified in the IPA pathway. In addition, the 26S proteasome was also identified in this pathway. It was demonstrated that Golgi membrane protein, RER1, specifically mediates aSyn degradation through the ubiquitin-proteasome system (Park et al., 2017). Based on both current literature and IPA analysis, the proteasome and lysosome are important in aSyn degradation. Therefore, the failure of aSyn interactions with both degradation pathway components contributes to increasing aSyn pathology.

## Conclusion

IPA and literature analysis conducted in this work identified a network of possible interacting partners of aSyn. Predicted aSyn interacting proteins include chaperones/chaperonins, translocating factors, modifying enzymes, mitochondrial-associated proteins, and proteins involved in secretion and degradation. Disruptions in these interactions can result in a change of aSyn biogenesis, leading to its aggregation and subsequent pathology. We propose that alterations of these interactions are key elements for the molecular mechanism of aSyn aggregation and associated pathology in PD. Modulation of these interacting partners may prevent aSyn aggregating and reverse the damaging effects in synucleinopathies, thus opening new directions in pharmacological targets for the disease treatment.

## Author Contributions

AK proposed conceptual idea and coordinated the project. SH and AK wrote the manuscript. SH and ET designed and prepared figures. SH conducted analysis by the use of IPA. ET provided corrections and critical review of the manuscript. All authors discussed and edited the manuscript.

## Conflict of Interest

The authors declare that the research was conducted in the absence of any commercial or financial relationships that could be construed as a potential conflict of interest.
